# Incidental Findings on Brain MR Imaging in Older Community-Dwelling Subjects Are Common but Serious Medical Consequences Are Rare: A Cohort Study

**DOI:** 10.1371/journal.pone.0071467

**Published:** 2013-08-15

**Authors:** Elaine M. Sandeman, Maria del Carmen Valdes Hernandez, Zoe Morris, Mark E. Bastin, Catherine Murray, Alan J. Gow, Janie Corley, Ross Henderson, Ian J. Deary, John M. Starr, Joanna M. Wardlaw

**Affiliations:** 1 Brain Research Imaging Centre, Division of Clinical Neurosciences, University of Edinburgh, Edinburgh, United Kingdom; 2 Centre for Cognitive Ageing and Cognitive Epidemiology, University of Edinburgh, Edinburgh, United Kingdom; 3 Scottish Imaging Network, A Platform for Scientific Excellence (SINAPSE) Collaboration, Department of Clinical Neurosciences, The University of Edinburgh, Edinburgh, United Kingdom; 4 Department of Psychology, University of Edinburgh, Edinburgh, United Kingdom; 5 Department of Medicine for the Elderly Western General Hospital, Edinburgh, United Kingdom; 6 Alzheimer Scotland Dementia Research Centre, University of Edinburgh, Edinburgh, United Kingdom; Emory University, United States of America

## Abstract

**Objectives:**

Incidental findings in neuroimaging occur in 3% of volunteers. Most data come from young subjects. Data on their occurrence in older subjects and their medical, lifestyle and financial consequences are lacking. We determined the prevalence and medical consequences of incidental findings found in community-dwelling older subjects on brain magnetic resonance imaging.

**Design:**

Prospective cohort observational study.

**Setting:**

Single centre study with input from secondary care.

**Participants:**

Lothian Birth Cohort 1936, a study of cognitive ageing.

**Main Outcome Measures:**

Incidental findings identified by two consultant neuroradiologists on structural brain magnetic resonance imaging at age 73 years; resulting medical referrals and interventions.

**Primary and Secondary Outcome Measures:**

Prevalence of incidental findings by individual categories: neoplasms, cysts, vascular lesions, developmental, ear, nose or throat anomalies, by intra- and extracranial location; visual rating of white matter hyperintensities and brain atrophy.

**Results:**

There were 281 incidental findings in 223 (32%) of 700 subjects, including 14 intra- or extracranial neoplasms (2%), 15 intracranial vascular anomalies (2%), and 137 infarcts or haemorrhages (20%). Additionally, 153 had moderate/severe deep white matter hyperintensities (22%) and 176 had cerebral atrophy at, or above, the upper limit of normal (25%) compared with a normative population template. The incidental findings were unrelated to white matter hyperintensities or atrophy; about a third of subjects had both incidental findings and moderate or severe WMH and a quarter had incidental findings and atrophy. The incidental findings resulted in one urgent and nine non-urgent referrals for further medical assessment, but ultimately in no new treatments.

**Conclusions:**

In community-dwelling older subjects, incidental findings, including white matter hyperintensities and atrophy, were common. However, many findings were not of medical importance and, in this age group, most did not result in further assessment and none in change of treatment.

## Introduction

In neuroimaging research, incidental findings are defined as “apparently asymptomatic intracranial abnormalities that are clinically significant because of their potential to cause symptoms or need to be treated” [1], [2]. They are unknown to the subject and unrelated to the purpose of the imaging. They may cause anxiety, and potentially have medical, lifestyle or financial consequences [3]. They are an increasing problem in imaging research, in health screening and in clinical practice.

Most data on the frequency of incidental findings in neuroimaging come from young subjects [4]. In a meta-analysis of 16 neuroimaging studies including 19,559 volunteers with mean ages from 11 to 63 years examined with MRI, the overall prevalence of incidental findings was 3%, giving a “number needed to scan” of 37 to detect any incidental finding [4]. The incidence increased with age and magnet field strength. The results were of limited relevance to the ageing population, however, as few subjects were aged over 70 years, there existed between-study heterogeneity for some findings, and many findings were poorly described.

There is no systematic assessment of the medical or non-medical consequences of incidental findings, only anecdotal or retrospective reports [5], with no reliable data on subsequent treatment or lifestyle impact. Anecdotal reports indicate that incidental findings may cause considerable anxiety to the individual [6], [7], plus nuisance to individuals and medical services [7]. If arising during investigation for another illness, they may deflect attention away from important primary health findings [7], [8]. In research or commercial screening they may inflate health care costs and workload of already stretched medical services [9].

People are living longer. By 2050, 40% of populations in many Western countries will be over 50 and 25% over 65. Promotion of lifelong health and wellbeing is a government research priority in which imaging of brain structure and function plays a major role [10]. The paucity of data on the frequency, medical and lifestyle implications of incidental findings in neuroimaging in older people makes it difficult to inform subjects accurately about participating in research, particularly at ages when factors such as access to travel and other types of health-related insurance may already be difficult. Companies offering scanning as part of ‘lifestyle health screening’ often target an older clientele [11].

We examined brain MRI data acquired from community-dwelling older subjects during research on cognitive ageing to determine the age-related prevalence and medical consequences of key incidental findings.

## Methods

### Subjects

Subjects were participants of the Lothian Birth Cohort 1936 (LBC1936), a longitudinal study of cognitive ageing. This cohort includes 1091 community-dwelling adults most of whom completed the Moray House Test (MHT) of verbal reasoning as part of the Scottish Mental Survey 1947 (SMS1947) at a mean age of 11 years [12]. All of the LBC1936 participants were born in 1936 and most resided in the Edinburgh area of Scotland when recruited at age 70 years. In the first wave of the study, the 1091 LBC 1936 subjects were retested on the MHT in addition to other detailed cognitive, sociodemographic and physical assessments. Around three years later, at about age 73 years, all surviving members who were non-demented were invited for re-testing and 866 members of the cohort returned for re-testing (second wave) this time including detailed structural brain MRI [13]. Subjects with possible dementia (Mini Mental State Exam (MMSE) scores <24 [14]) were excluded. The study was approved by the Lothian (REC 07/MRE00/58) and Scottish Multicentre (MREC/01/0/56) Research Ethics Committees and all subjects gave written, informed consent.

### Neuroimaging

Participants underwent brain MRI using a GE Signa Horizon 1.5 T HDxt clinical scanner (General Electric, Milwaukee, USA) equipped with a self-shielding gradient set (33 mT m^−1^ maximum gradient strength) and manufacturer supplied 8-channel phased-array head coil. The examination comprised whole brain T_2_-, T_2_*- and FLAIR-weighted axial sequences, a high-resolution T1-weighted volume scan acquired in the coronal plane from the upper neck to vertex, and diffusion tensor (DT), magnetization transfer (MT) and T1-mapping brain MRI sequences [13].

### Radiological Reporting

The structural MRI data (axial T2, T2*, FLAIR, coronal T1) were transferred to the Scottish National Picture Archiving and Communications System (PACS) and reported by a consultant neuroradiologist (JMW) blind to all information about the subjects except for their participation in the study. A second neuroradiologist (ZM) also evaluated the images independently. Information from the two sources was combined and any discrepancies discussed. All lesions were identified according to standard clinical neuroradiological practice based on their typical appearances, e.g. of neoplasms or arteriovenous malformations, aneurysms, infarcts (cortical, subcortical or posterior fossa) or haemorrhages, arachnoid cysts, etc. White matter hyperintensities (WMH) were scored using the Fazekas scale, a well validated visual rating method which codes the deep and periventricular lesions separately, each on a scale of zero to three [15]. WMH were dichotomised into none/mild (Fazekas 0, 1) and moderate/severe (Fazekas 2, 3). Brain atrophy was coded in six categories by comparison with a validated visual template derived from normal subjects aged between 65–70 and 75–80 years in our population [16], which codes superficial and deep atrophy separately on a six point scale (‘5’ represents the upper 95% confidence interval and ‘6′ indicates brain volume loss in excess of normal for age).

### Medical Assessment

The radiological reports were reviewed by a consultant geriatrician (JMS) with access to the subjects’ hospital medical records, the subject’s General Practitioner and the subject themselves and who could act on any findings. Findings were discussed by the radiologists and geriatrician and other relevant clinicians as necessary, including neurologists and stroke physicians, and referred to the subject’s family doctor when appropriate.

### Analysis

Any structural incidental findings were categorised into pre-specified intra- or extra-cranial pathological categories: neoplasms, cysts, vessel abnormalities, infarcts or haemorrhages, ENT problems, structural variants, and others. The WMH and atrophy ratings initially were analysed separately from the counting of structural incidental findings as WMH and atrophy are common accompaniments of ageing for which at present the causes are poorly understood and there is no proven treatment. Additionally, the WMH [15] and atrophy [16] quantification were performed using established scales detailed above to improve consistency and specificity rather than using the verbal descriptions of WMH and atrophy in radiological reports. We then examined the proportion of subjects with incidental findings by WMH score (summed Fazekas score of 0 to 6) and by deep and superficial atrophy scores at or above the upper limit of normal for age according to our population normal template. The number of participants referred for further assessment or treatment and any resulting action (urgent, non-urgent, none) was assessed by cross-referencing with the case notes and hospital records.

## Results

Of the 741 participants who agreed to undergo brain MRI, 700 (mean age 72.5 years; SD 1.5 years; 368 male) provided usable structural MRI images. Subjects unable to provide MRI data included 29 with claustrophobia, four whose physical size or shape precluded lying in the scanner, four who were too unwell to be scanned, three with dental artefact problems, and one with a stapedectomy (contraindication to MRI).

**Figure 1:**
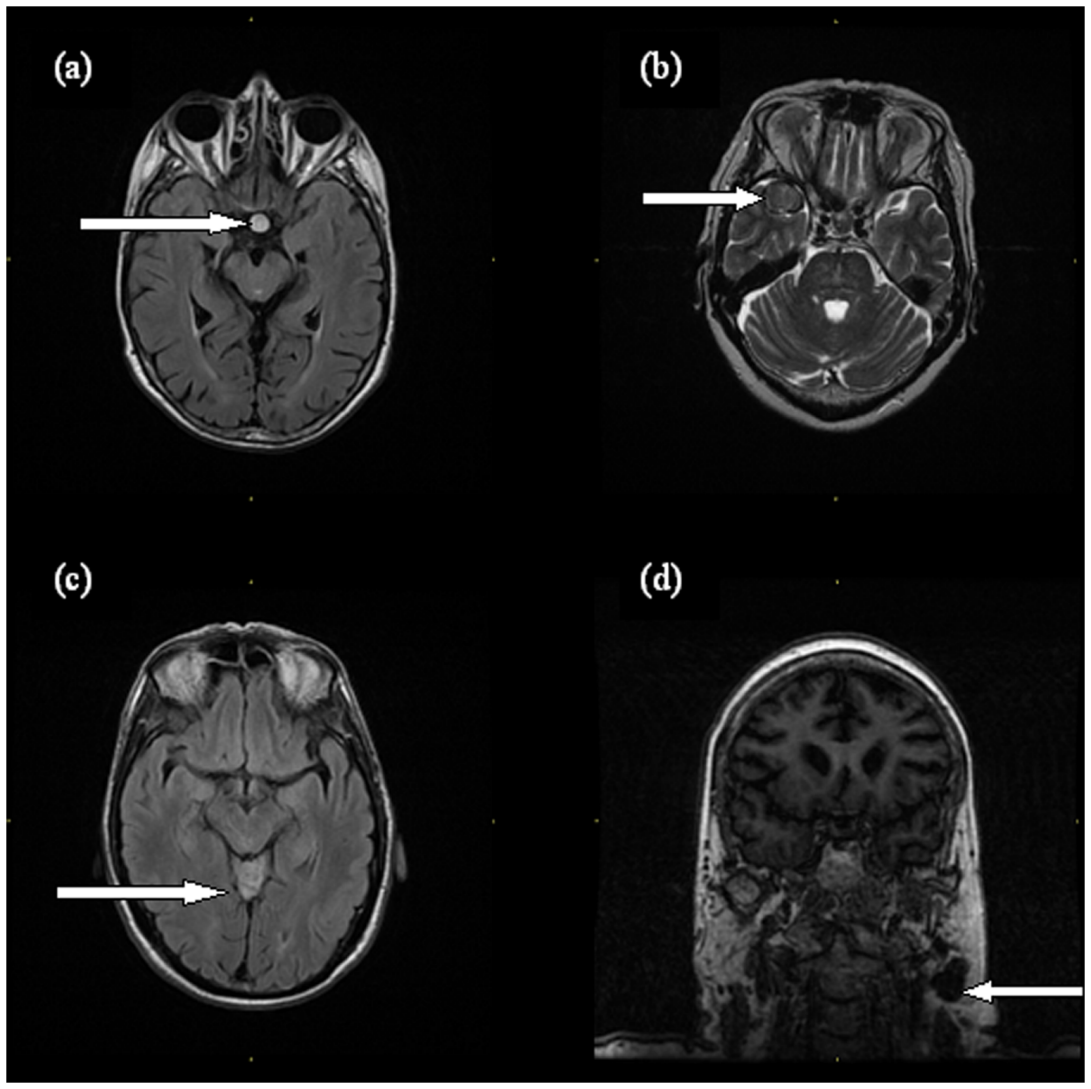
Examples of incidental findings in the LBC1936: (a) pituitary adenoma, (b) giant middle cerebral artery aneurysm, (c) Lhermitte-Duclos disease and (d) pleomorphic salivary adenoma.

Of the 700 subjects with usable brain images, 223 (32%, 95% CI 28–35%), of whom 89 were female and 134 were male, had 281 incidental findings (Table 1); 172 subjects had a single finding, 44 had two, and seven had three. Examples of incidental findings can be seen in Figure 1.

**Table 1:** Total number and type of incidental findings in 223 subjects.

Lesion	Lesion type	Total*	Urgent action	Routine action	No action
Neoplasms					
	Intracranial				
	Menigiomas+	5		1	4
	Pituitary+	2		2	0
	Cerebellar tumour+	1		1	0
	Arachnoid/cystic neoplasm+	1		1	0
	Enlarged choroid plexus	1			1
	Extracranial				
	Salivary adenoma+	3		2	1
	Lipoma on neck	1			1
	**TOTAL**	**14**		**7**	**7**
Cysts§					
	Arachnoid cysts	10			10
	Pituitary cysts	1			1
	Other cysts				
	ENT related	1			1
	CSF cyst	1			1
	Choroidal fissure cyst	1			1
	Soft tissue cyst	1			1
	**TOTAL**	**15**			**15**
**Vascular-related abnormalities (except stroke lesions – see below)**					
	Aneurysm+	5	1	1	3
	Cavernous haemangioma	3			3
	Subdural hygroma+	1			1
	Occluded ICA	2			2
	Venous angioma	2			2
	Basal arterial ectasia	1			1
	**TOTAL**	**14**	**1**	**1**	**12**
Stroke Lesions (see Table 2 for further detail)					
	Old infarction	72			72
	Old haemorrhage	7			7
	Micro haemorrhage	39			39
	Lacunes	14			14
	Superficial siderosis	1			1
	Infarct-related cerebromalacia	2			2
	Central pontine hyperintensity	1			1
	Increased iron deposition – no micro haemorrhage	1			1
	**TOTAL**	**137**			**137**
ENT problems					
	Sinus problems	62			62
	Mastoid problems+	6		1	5
	Maxillary polyps	6			6
	ENT developmental	2			2
	**TOTAL**	**76**		**1**	**75**
Structural variants					
	Asymmetry of cerebral hemispheres	2			2
	Left temporal atrophy	1			1
	Normal variant septum pellucidum	4			4
	Chiari I malformation	1			1
	Calcification focal iron deposits	1			1
	Developmental variation in sulcation of cerebellum	1			1
	Atrophic cerebellum	1			1
	Calcifications	6			6
	**TOTAL**	**17**			**17**
Others					
	Congenital arch of C1 vestigial	1			1
	Eye problems	2			2
	Previous Surgery	2			2
	Artefact	3			3
	**TOTAL**	**8**			**8**
**TOTAL**		**281**	**1**	**9**	**271**

* 172 had one, 44 with two and seven with three findings (total 281 findings).

+ considered of potential health consequence;

§ note several of these if large or if confused with other pathologies could also be considered ‘of health consequence’.

There were 14 intra- or extracranial tumours (2% of the 700 subjects, 95% CI 1.1–3.3%), 15 developmental or acquired cysts (2.4%, 95% CI 1.2–3.5%), 14 vascular abnormalities (2%, 95% CI 1.1–3.3%), 76 varied ENT problems (10.9%, 95% CI 8.6–13.2%), and 16 other varied findings including other developmental variants (2.3%, 95% CI 1.3–3.4%). Eighty-six subjects had one or more infarcts most of which were very small and included lacunes (12%, 95% CI 10–15%), 39 subjects had one or more microhaemorrhages (6%, 95% CI 4–8%), and seven subjects had one or more old primary haematomas all small (1%, 95% CI 0.4–2%: further details in Table 2).

**Table 2:** Number of subjects with several of the same type of finding within the stroke lesion section of Table 1.

Category	Subcategory	Number of subjects
Old infarction		
	Total with one or more	72
	With 2	16
	With 3	4
	With 4	1
	With several	4
Lacunes		
	Total with one or more	14
	With 2	1
	With several	7
Micro haemorrhage		
	Total with one or more	39
	With 2	4
	With 3	4
	With several	8

Moderate periventricular WMH were present in 184/700 (26%) and deep WMH in 130/700 (18%,) and severe periventricular WMH were present in 46/700 (6%) and deep WMH in 23/700 (3%), Table 3; 129/70 (18%) had deep and 149/700 (21%) had superficial atrophy at the upper limit of normal for age; 45/70 (6%) had deep and 27/700 (4%) had superficial atrophy above the upper limit of normal for age (Table 4). There was no association between incidental findings and WMH or atrophy scores (Table 5), the number of subjects with incidental findings and moderate to severe WMH scores (83/223, 37%) or atrophy above the upper limit of normal (61/223, 27%, with deep atrophy) being the same as in those without incidental findings (35% and 23% respectively).

**Table 3:** The proportion of subjects by Fazekas visual rating score.

Scores	Percentage of subjects	
	Periventricular	Deep white matter
0	25 (3.2%)	109 (15.5%)
1	445 (64.2%)	438 (63.2%)
2	184 (26.3%)	130 (18.4%)
3	46 (6.3%)	23 (2.9%)

**Table 4:** The proportion of subjects with each atrophy visual rating scores.

Atrophy scores	Percentage of subjects	
	Deep atrophy	Superficial atrophy
1	50 (7.0%)	46 (6.4%)
2	88 (12.4%)	92 (13.0%)
3	219 (31.5%)	212 (30.5%)
4	170 (24.4%)	175 (25.1%)
5	129 (18.4%)	149 (21.3%)
6	45 (6.3%)	27 (3.7%)`

**Table 5:** Number (%) of subjects with incidental findings (IF), WMH according to visual rating by Fazekas score and atrophy against a visual population normal for age template. Fazekas total score = sum of deep and periventricular WMH scores.

Scale	WMH Fazekas total score			Atrophy deep			Atrophy superficial		
	IF	No IF	Whole cohort	IF	No IF	Whole cohort	IF	No IF	Whole cohort
0	0 (0%)	11 (2.4%)	11 (1.6%)	–			–		
1	32 (14.5%)	74 (15.9%)	106 (15.5%)	16 (7.2%)	32 (6.9%)	48 (7.0%)	12 (5.4%)	32 (6.9%)	44 (6.4%)
2	106 (48.0%)	214 (46.1%)	320 (46.7%)	18 (8.1%)	67 (14.4%)	85 (12.4%)	21 (9.5%)	68 (14.7%)	89 (13.0%)
3	**47 (21.3%)**	**91 (19.6%)**	**138 (20.1%)**	67 (30.3%)	149 (32.1%)	216 (31.5%)	61 (27.6%)	148 (31.9%)	209 (30.5%)
4	**24 (10.9%)**	**45 (9.7%)**	**69 (10.1%)**	59 (26.7%)	108 (23.3%)	167 (24.4%)	59 (26.7%)	113 (24.4%)	172 (25.1%)
5	**8 (3.6%)**	**20 (4.2%)**	**28 (4.1%)**	**41 (18.6%)**	**85 (18.3%)**	**126 (18.4%)**	**56 (25.3%)**	**90 (19.4%)**	**146 (21.3%)**
6	**4 (1.8%)**	**9 (1.9%)**	**13 (1.9%)**	**20 (9.0%)**	**23 (5.0%)**	**43 (6.3%)**	**12 (5.4%)**	**13 (2.8%)**	**25 (3.6%)**

Bold indicates WMH scores consistent with moderate to severe WMH and atrophy at or above the upper limit of normal for age. The scale indicates a) the summed deep (0–3) and periventricular (0–3) Fazekas scores = total WMH score of 0–6, and b) the atrophy scores where 1 = <25^th^ centile, 2 = 25–50^th^ centile, 3 = 50–75^th^ centile, 4 = 75–95^th^ centile, 5 = just above 95^th^ centile and 6 = considerably above the 95^th^ centile for age.

Amongst the structural findings, 18 (i.e., in 2.6% of the 700 participants) could be regarded as of potential health importance (tumours including salivary adenomas, aneurysms, subdural hygromas), whereas in this age group the arachnoid cysts and cavernous angiomas would generally not be. Similarly, asymptomatic infarcts, small haematomas, microbleeds, and other vascular lesions would also generally not result in clinical referral.

The findings lead to ten referrals (3 male, 7 female) by the study geriatrician for further specialist medical assessment (1.5% of the 700, or 4.4% of the 223 who had a structural incidental finding), of which one was referred urgently (giant middle cerebral artery aneurysm) and nine non-urgently (two pituitary tumours, two salivary adenomas, one left middle cerebral artery aneurysm, one meningioma, one cerebellar tumour, one arachnoid/cystic neoplasm and one mastoid problem). The remaining 271 findings (96%) in 213 subjects (96%), including the remaining 8/18 findings that could be regarded as of potential health importance, were judged to require no further action.

Amongst the ten subjects referred for further medical assessment by the geriatrician, the one subject who was referred urgently because of a giant middle cerebral aneurysm had a cerebral angiogram which confirmed the MRI findings. However, this subject was completely asymptomatic and declined further treatment. The other patient with a left middle cerebral artery aneurysm underwent MR angiography that confirmed the aneurysm but this was considered low risk for bleeding based on size and medical risk factors so no further action was taken. One of the five meningiomas was referred for a further MRI with contrast which was performed 6 months after the index scan and showed no change. It was decided simply to observe the tumour thereafter. Of the two suspected pituitary tumours, one underwent detailed contrast-enhanced MRI which failed to confirm a tumour. The other had had no further imaging by the end of the present study period due to requiring treatment for diverticular disease which was deemed to be more urgent as there was no evidence of a visual field defect or hormonal imbalance on blood testing that could be attributed to the pituitary tumour. The two salivary adenomas and the subject with the mastoid abnormality were referred for further imaging; the salivary adenomas were deemed to be slow growing pleiomorphic ademomas that were too small to merit surgical removal and were kept under review; similarly the mastoid lesion was deemed to be longstanding and not requiring active intervention. The patient with the cerebellar tumour underwent further imaging and the lesion was deemed to be benign and thereafter was simply kept under review. The one arachnoid/cystic neoplasm was confirmed to be an arachnoid cyst after further imaging, ie a longstanding static developmental anomaly, that required no further action.

Of the remaining 8/18 findings that were of potential health importance, the remaining three aneurysms were all deemed too small for treatment, so no further action was taken. The other four meningiomas were small and required no follow up. One subdural hygroma was not associated with any symptoms, the subject was not on any drugs that would increase the risk of bleeding and the hygroma was not displacing the brain; therefore no further action was taken.

## Discussion

Incidental findings on neuroimaging, excluding WMH and cerebral atrophy, are common (32% of 700 participants) in older people, but most were clinically non-significant and did not result in, or require any, further medical action. This proportion with incidental findings is much higher than the summary estimate from a meta-analysis of all previous neuroimaging studies (3%) most of which studies included subjects who were much younger [4]. The prevalence of specific lesions such as neoplasms (2%) is similar. The prevalence of stroke lesions (20%) was higher. The higher overall prevalence may reflect the different categorisation of findings in previous studies as well as our inclusion of head and neck abnormalities, the older age of our subjects and our use of high definition scans. Adding in the proportion of subjects with incidental findings and moderate or severe WMH (about a third) or atrophy (about a quarter) still shows that many subjects in this age category have structural or aging related findings on brain imaging.

Most of the findings in the present study had little clinical consequence but could have generated a substantial volume of work, anxiety and non-medical consequences without neuroradiology and a physician geriatrician to avoid over-interpretation of minor abnormalities. At present, asymptomatic cerebrovascular lesions (infarcts, haemorrhages, lacunes, WMH, microbleeds) do not result in immediate medical referral or interventions. In this age group, many subjects are already taking preventive or other medicine prescribed for hypertension or other vascular disease, so no additional action was deemed necessary. This would not apply in a younger population. Of the few findings with potential for greater current clinical health impact (tumours, aneurysms), several were simply monitored with further imaging, and others were not investigated further as the subject was asymptomatic and there was no indication. The patient with the giant aneurysm that resulted in urgent assessment declined further treatment. Again, in a younger population, where tumours and aneurysms may be more aggressive, these findings could have resulted in more active medical intervention. As the present study population are part of a longitudinal cohort study, they will be reviewed every few years and this also may increase the tendency to ‘keep findings under review’ rather than to intervene more aggressively. Regardless, sensitive handling of potentially anxiety provoking findings is important to avoid causing undue distress, unjustifiably inflating the research, clinical workload and costs, and contributing unnecessarily to adverse medical and potential non-medical consequences.

About a quarter of subjects had moderate (26% periventricular and 18% deep) or severe (6% periventricular and 3% deep) WMH and a fifth of subjects had brain atrophy at (18% deep, 21% superficial) or above (6% deep, 4% superficial) the upper limit of normal for age as judged against an age-relevant normal template [16], as is common in this age group. We distinguished these features from the structural incidental findings as WMH and atrophy are best assessed using structured rating scales rather than with free text as in a standard radiological report. However the rating was by the same neuroradiologists. Loss of brain tissue is associated with advancing age and dementia, although all subjects had cognitive function tests that excluded dementia as a requirement for participation in the study. About a third of subjects had at least one incidental finding and moderate to severe WMH, and about a fifth of subjects have at least one incidental finding and atrophy above the upper limit of normal for age (Table 5). At present there is no specific treatment to avoid atrophy or prevent or reduce WMH and therefore no specific action was taken as a consequence of these findings. Clearly that would change should a treatment become available. The subjects’ vascular risk factors were assessed as part of the study and subjects with abnormal results (eg elevated blood pressure not already known about) were notified to the family doctor.

The study has limitations. Our results may not extrapolate directly to the medical consequences of incidental findings in younger people where some lesions may lead to more active intervention. The prevalence of WMH and atrophy would not apply in younger people. We did not obtain information on any non-medical consequences of the incidental findings such as financial, emotional, lifestyle consequences. The study is longitudinal and this may have lead to an expectation that any lesion progression could be assessed at follow-up imaging, which may have diminished the number of referrals for further assessment.

Our study has strengths. It is large (700 subjects). The structural sequences were of high quality and similar to those used for clinical MRI and all scans were examined by consultant neuroradiologists using a high quality clinical image viewing system. Both of these features may have increased the detection rate compared with, for example, functional MRI research, where typically few images with diagnostic relevance are obtained. We included head and neck abnormalities as these structures are commonly covered by brain volume images. We also provided information on medical impacts.

Three other studies examined incidental findings in people aged over 60. Vernooij et al. [17] examined 2000 people, mean age 63.3 years (range 45.7–96.7) in the Rotterdam Scan Study but did not provide a “per subject incidental finding rate” (only rate per type of finding): our rates for meningiomas, pituitary adenomas and arachnoid cysts were similar, but we found more aneurysms and infarcts, possibly because our subjects were about 10 years older. Yue et al [18]. imaged 3672 subjects aged over 65 years in the Cardiovascular Health Study but only reported on potentially clinically serious lesions; therefore, only meningiomas, aneurysms and pituitary adenomas can be compared with the current study. Yue [18] found fewer of these lesions in 3672 subjects aged over 65 in the Cardiovascular Health Study than either Vernooij et al. or the present study, although differences in scanning protocols, use of a very low field strength scanner at one center (0.35 Tesla) with low image resolution, and inclusion of less extracranial tissue in the head image, may partly explain these differences [4]. Yue et al considered meningiomas (19, of which eight were larger than 2.5 cm diameter), aneurysms (4, of which one was giant), cavernous malformations (5, which were multiple in one subject), subdural collections (2), vascular stenosis (8), various other tumours including pituitary tumours (10), extracranial (8) and other miscellaneous lesions (8) as meriting ‘immediate’ or ‘urgent’ medical alerts, a total of 64/3672 subjects affected or 1.74%. Some of these (eg the largest meningioma and the giant aneurysm) were known to the subjects prior to scanning, so were not truly ‘incidental’. The proportion is similar to the proportion of our subjects (18/700, 2.6%) with these lesion types, although if we also include the arterial occlusions, cavernous malformations and other intra- and extracranial lumps that we did not consider to require further action (but Yue et al did), then the proportion of these lesion types rises to 30/700 (4.3%) in our population. The only study that did provide systematic information on medical consequences was retrospective and based on older technology (published in 1999 but the study occurred between 1996 and 1997): 18% of 1000 healthy volunteers aged 3–83 years participating in various research projects had an abnormal scan, of which 15% were not referred, 2% were referred for routine assessment and 1% were referred for urgent assessment [5]. One study of 206 young healthy volunteers published since the completion of the systematic review found incidental abnormalities in 19% (of which about half were of potential clinical relevance), but provided no data on the impact or consequences of the findings themselves [19].

‘Incidentalomas’ are an increasing, but not new problem with greater use of MRI for research and in the public and private health care sectors [8], [20]. Volunteers in research are essential. There is little research assessing subjects’ own awareness of potential incidental findings or their management [3], [21], although most potential research volunteers with [21] or without [22] personal experience of research imaging indicate wished to be informed of incidental findings that might have likely health consequences. Much neuroimaging research, especially that involving advanced imaging like functional MRI, is conducted by researchers without clinical training in how to interpret MRI data [23], [24]. Incidental findings may be even more common in body imaging, although even fewer data are available than for brain imaging [25]. Following widespread consultation amongst imaging research centres, professional organisations, research funding agencies, ethicists and lay people (including debate at the UK Biobank Ethics and Governance Council [20]) the UK now has published guidance on minimum standards for the “*Management of incidental findings detection during research imaging*” (http://www.rcr.ac.uk/docs/radiology/pdf/BFCR(11)8_Ethics.pdf) [3], and a recent report of volunteers’ opinions [22]. Volunteers’ opinions indicate the need for sensitive, proportionate and effective mechanisms for the routine management of incidental findings to avoid undue anxiety, particularly when any incidental finding can occur in such a large proportion (a third) of the volunteers.

The impact of incidental findings on employment, health, travel or life insurance, quality of life and medical costs is currently unknown. Similar problems regarding what to do about incidental findings in research using genetic and laboratory techniques are now being recognised [9], [20]. Future studies should focus on determining the medical and associated implications [7], including of treatment and outcome, of incidental findings in brain and body imaging research at all ages and in particular the non-medical implications and their impact on volunteers.
